# Supplementary material for: Multimodal epigenetic and enhancer network remodeling shape the transcriptional landscape of human beige adipocytes
Source: Commun Biol. 2026 Jan 8;9:191. doi: 10.1038/s42003-025-09469-8 (PMC12881478; doi:10.1038/s42003-025-09469-8)
Supplement: Supplementary file 2 — Supplementary Information [file 42003_2025_9469_MOESM2_ESM.pdf]

Multimodal epigenetic and enhancer network remodeling shape the transcriptional landscape of human beige adipocytes

Sarah Hazell Pickering[1§], Natalia M. Galigniana[1,3§], Mohamed Abdelhalim[1§], Anita L. Sørensen[1], Julia Madsen Østerbye[1], Manuela Zucknick[2], Philippe Collas[1,3*], Nolwenn Briand[1*]

[1]Department of Molecular Medicine, Institute of Basic Medical Sciences, Faculty of Medicine, University of Oslo, 0317 Oslo, Norway

[2]Oslo Centre for Biostatistics and Epidemiology, Institute of Basic Medical Sciences, Faculty of Medicine, University of Oslo, 0317 Oslo, Norway

[3]Department of Immunology and Transfusion Medicine, Oslo University Hospital, 0372 Oslo, Norway.

§Equal contribution

*Correspondence: philc@medisin.uio.no; nolwenn.briand@medisin.uio.no

Sarah Hazell Pickering: s.h.pickering@medisin.uio.no

Natalia M. Galigniana: n.m.galigniana@medisin.uio.no

Mohamed Abdelhalim: m.i.m.abdelhalim@medisin.uio.no

Anita Løvstad Sørensen: a.l.sorensen@medisin.uio.no

Julia Madsen Østerbye: j.k.madsen-osterbye@medisin.uio.no

Manuela Zucknick: manuela.zucknick@medisin.uio.no

Philippe Collas: philc@medisin.uio.no

Nolwenn Briand: nolwenn.briand@medisin.uio.no

Supplementary Figures

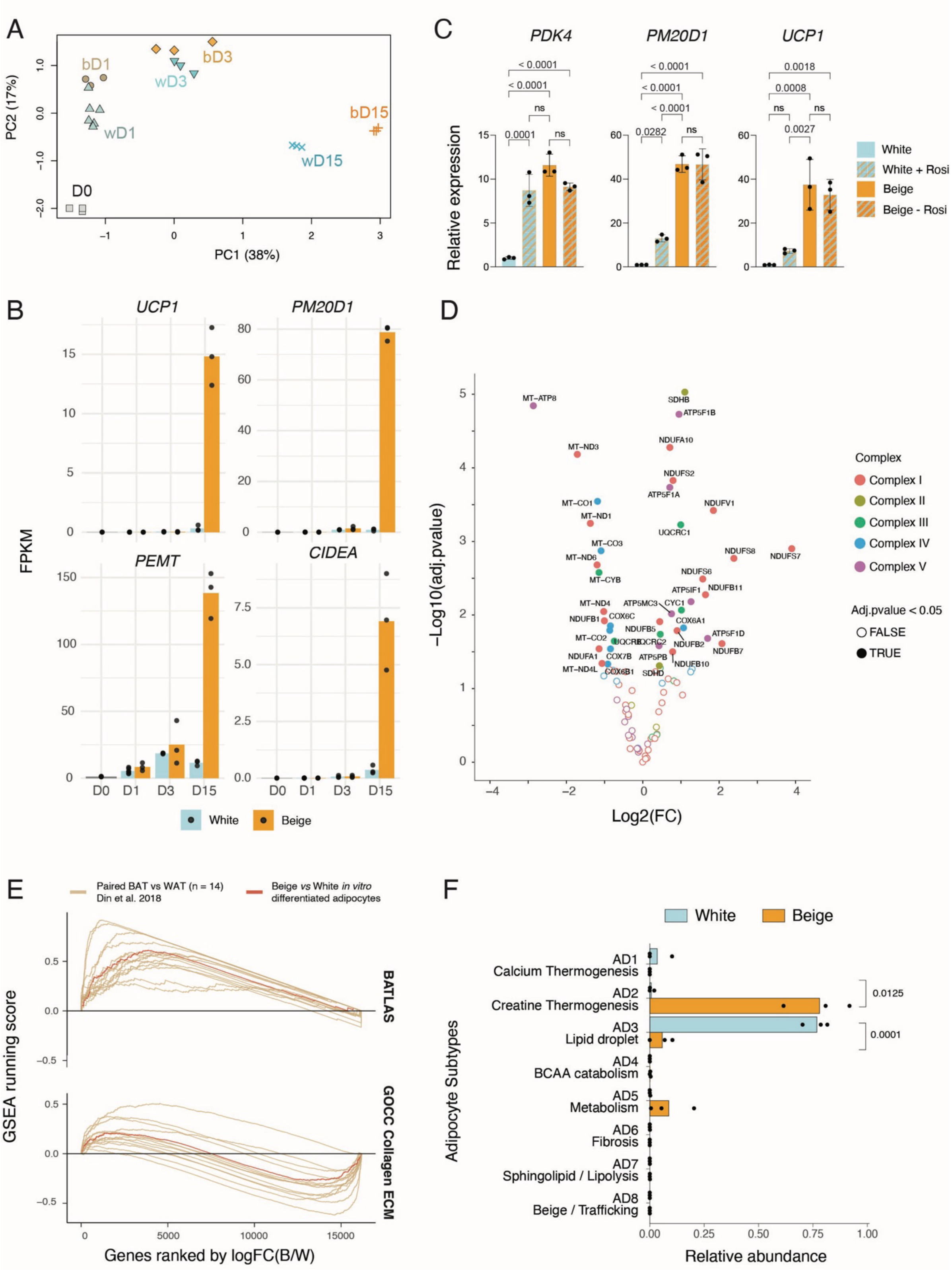

Supplementary Fig. 1. Related to Fig. 1. **A** Principal component analysis of time course RNA-seq. **B** Expression level (FPKM) of beige adipocyte markers during the differentiation time-course. **C** RT-qPCR analysis of *PDK4*, *PM20D1* and *UCP1* gene expression in white adipocytes ± 24 h 1 µM Rosiglitazone treatment and beige adipocytes ± 24 h Rosiglitazone withdrawal (mean ± SD; one-way ANOVA with Tukey's multiple comparisons test, ns non-significant; n = 3 independent differentiations). **D** Volcano plot of differential expression of genes from the mitochondrial respiratory chain in differentiated (D15) white and beige adipocytes. **E** Gene set enrichment analysis (GSEA) of paired human brown adipose tissue (BAT) vs white adipose tissue (WAT) and beige vs white in vitro differentiated adipocytes using BATLAS brown markers (top) and the gene ontology cellular component (GOCC) "collagen ECM" (bottom) gene signatures. **F** Estimated abundance of in vivo adipocyte subtypes (Miranda et al. 2025) in day 15 (D15) white and beige adipocytes based on deconvolution of RNA-seq (n = 3 independent differentiations; t-test).

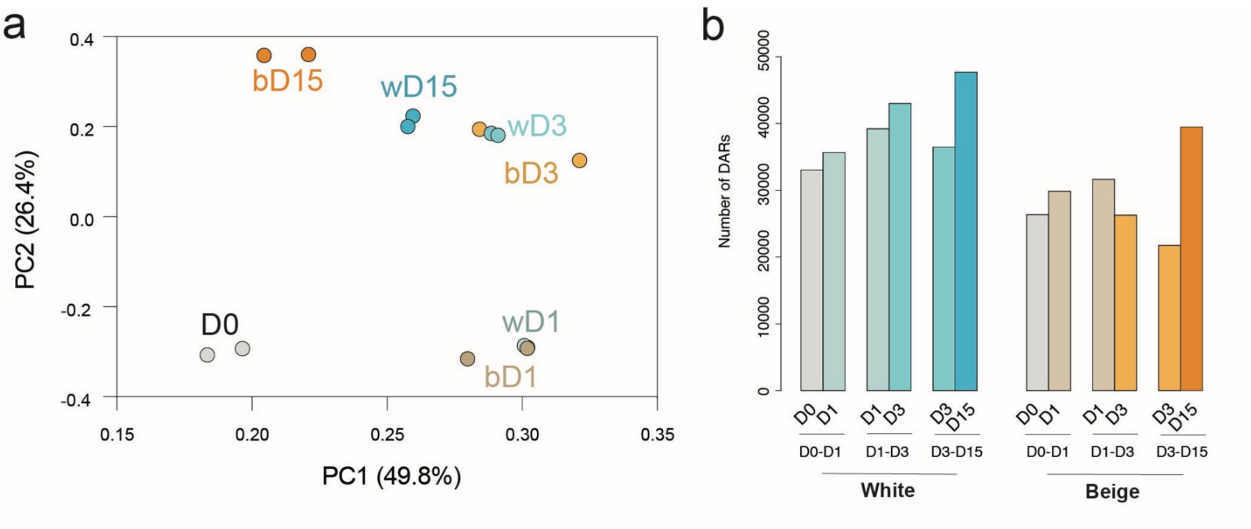

Supplementary Fig. 2. Related to Fig.2. **A** principal component analysis of time course ATAC-seq. **B** Number of DARs across the time course.

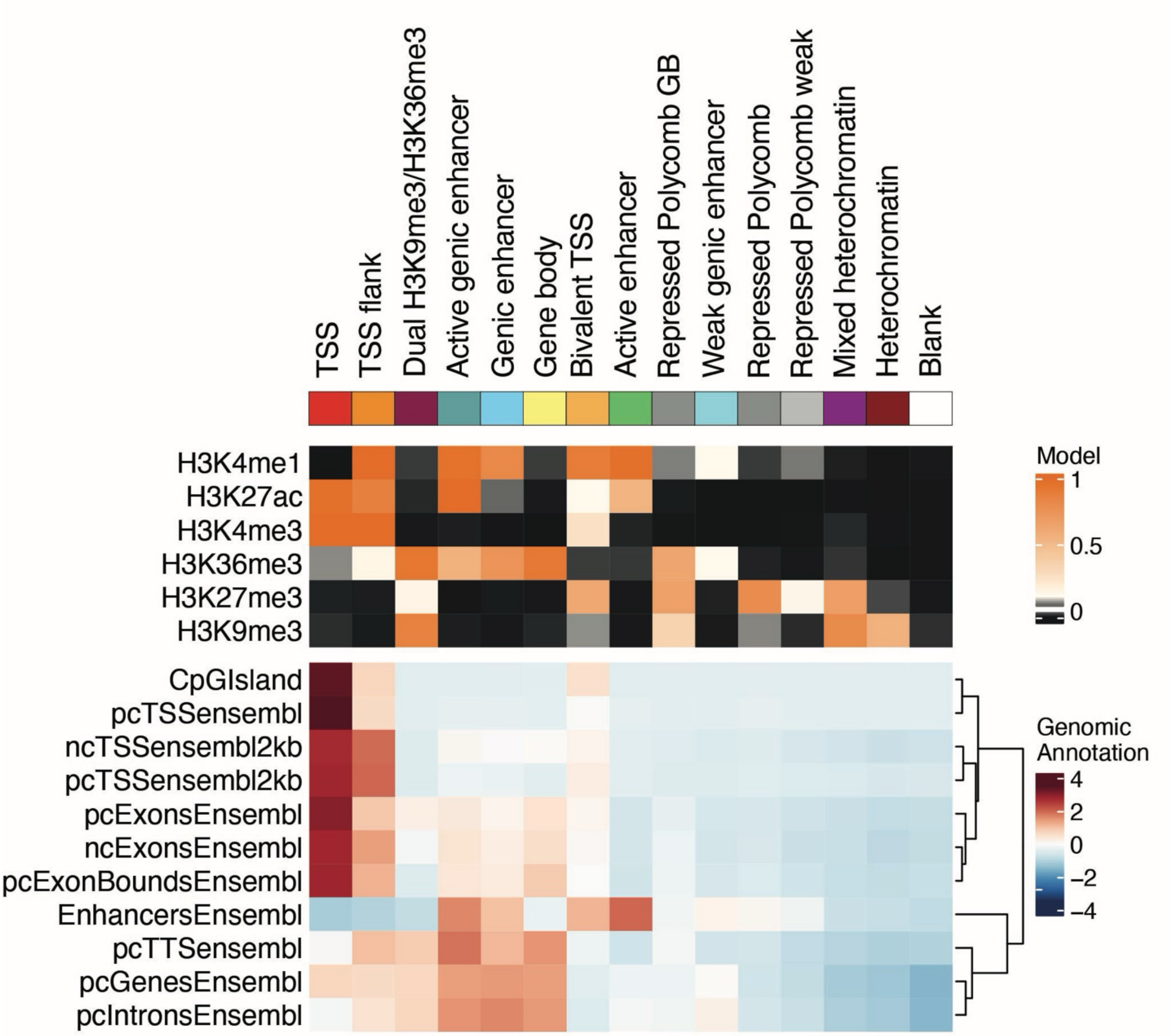

Supplementary Fig. 3. Related to Fig. 2. Emissions from the 15-state chromatin state model (top panel) and corresponding genomic annotation (lower panel)

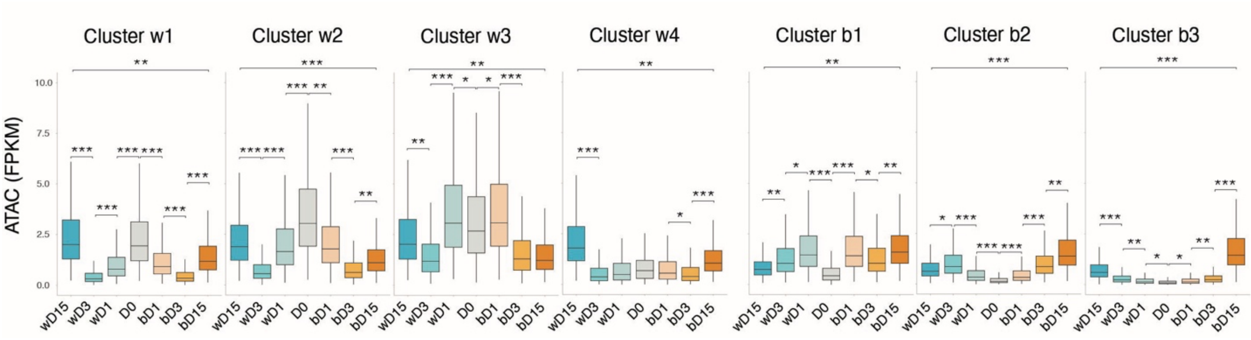

Supplementary Fig. 4. Related to Fig. 2. Quantification of ATAC signals within clusters (***| D | > 0.8, **| D | > 0.5, *| D | > 0.2; Cohen's D standardized mean difference).

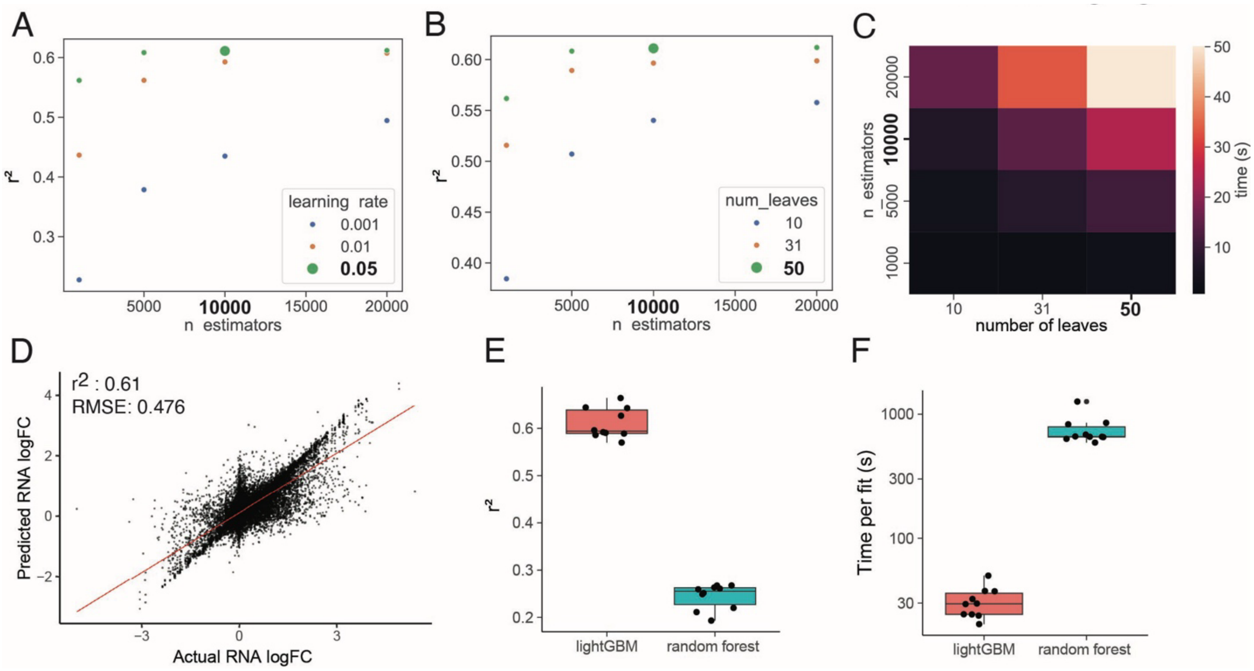

Supplementary Fig. 5. Related to Fig. 3. **A** Average model performance (r^2) for lightGBM models with different numbers of estimators and learning rates or **B** maximum number of leaves. Selected hyperparameter values are highlighted. **C** Average time taken to fit lightGBM models with different numbers of estimators and maximum number of leaves. **D** Scatter plot of actual vs predicted RNA log2 fold change of model trained with the best hyperparameters with a linear trendline. **E** Comparison of lightGBM and random forest (k=10) model performance and **F** time taken to fit. RMSE: root mean squared error.

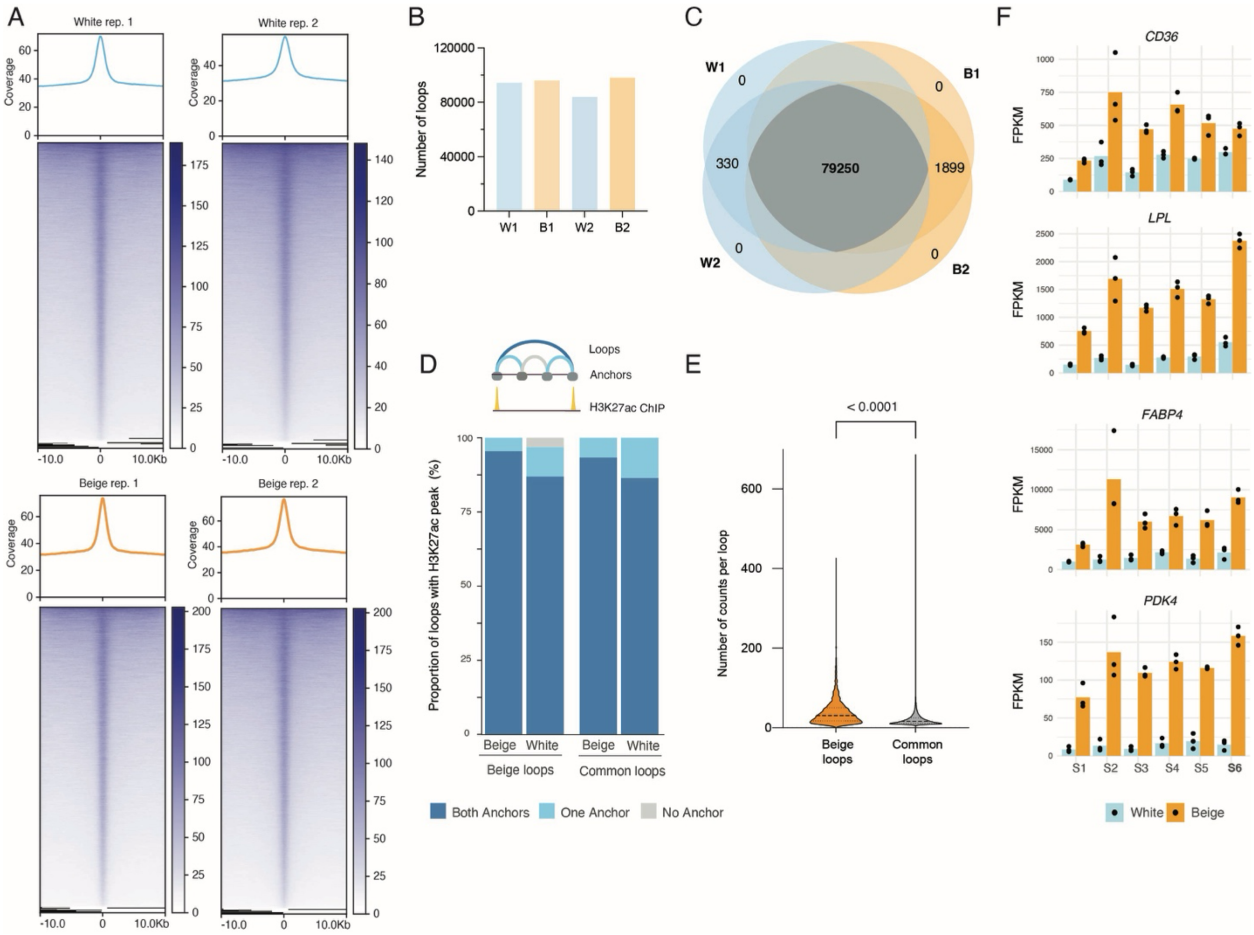

Supplementary Fig. 6. Related to Fig. 4. **A** Hi-ChIP signal enrichment at ChIP peaks per condition (White/Beige) and per replicate. **B** Number of called loops per replicate (≥ 6 counts). **C** Euler diagram showing overlap of called loops for each replicate. **D** Proportion of loop anchors overlapping with white or beige H3K27ac peaks from an independent H3K27ac ChIP-seq experiment. **E** Number of counts per loop in beige and common loops (two-tailed Mann-Whitney test; dashed lines represent the median and quartiles). **F** Expression level (FPKM) of differentially expressed genes ($p < 0.01$) with increased H3K27ac Hi-ChIP loops in white and beige adipocytes derived from the donor used in this study (S6), as well as from five unrelated human subjects (S1-5) [17].

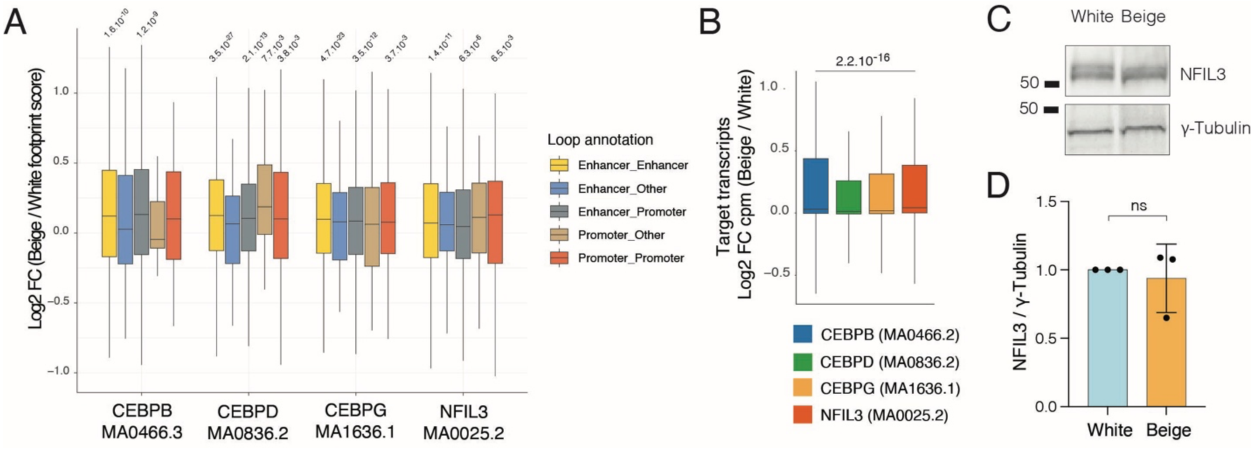

Supplementary Fig. 7. Related to Fig. 5. **A** Log2 fold change footprint scores per loop category for the indicated TF motifs (* $p < 0.01$, *** $p < 0.0001$, Wilcoxon signed-rank test) . **B** Log2 fold change beige vs white expression (normalized CPM over 300 bp downstream TSS) of transcripts with detected binding of indicated TFs at the promoter region (*** $p < 0.0001$, Wilcoxon signed-ranked test). **C** Western blot analysis and **D** quantification of NFIL3 expression in white and beige adipocytes (D15) (mean fold difference ± SD; ns non-significant, two-tailed paired Student's t test; n = 3). γ-Tubulin is shown as a loading control.

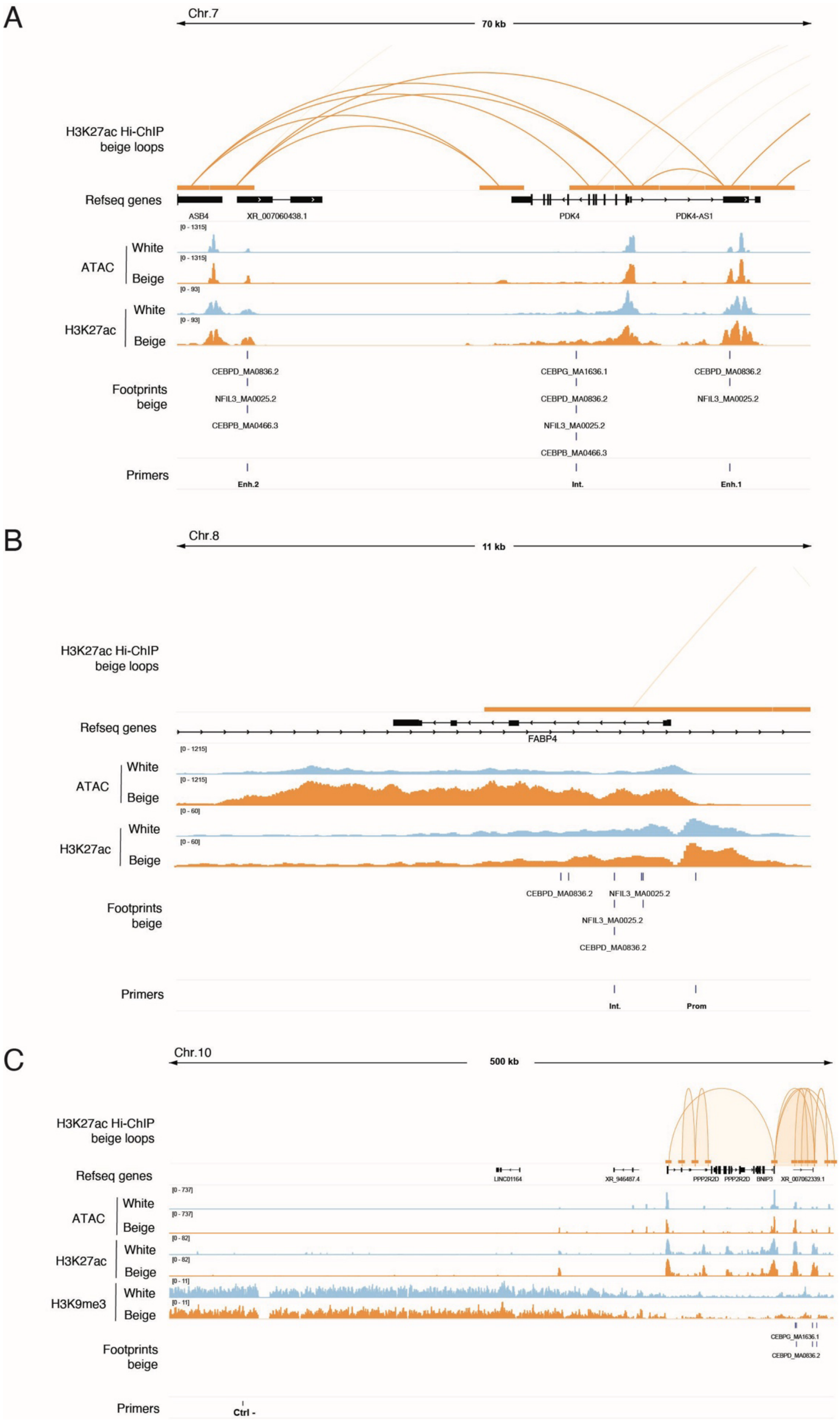

A
Chr.7
70 kb
H3K27ac Hi-ChIP beige loops
Refseq genes
ASB4
XR_007060438.1
PDK4
PDK4-AS1
ATAC
White
Beige
H3K27ac
White
Beige
[0 - 1315]
[0 - 93]
Footprints beige
CEBPD_MA0836.2
NFIL3_MA0025.2
CEBPB_MA0466.3
CEBPG_MA1636.1
CEBPD_MA0836.2
NFIL3_MA0025.2
CEBPB_MA0466.3
CEBPD_MA0836.2
NFIL3_MA0025.2
Primers
Enh.2
Int.
Enh.1
B
Chr.8
11 kb
H3K27ac Hi-ChIP beige loops
Refseq genes
FABP4
ATAC
White
Beige
[0 - 1215]
H3K27ac
White
Beige
[0 - 60]
Footprints beige
CEBPD_MA0836.2
NFIL3_MA0025.2
NFIL3_MA0025.2
CEBPD_MA0836.2
Primers
Int.
Prom
C
Chr.10
500 kb
H3K27ac Hi-ChIP beige loops
Refseq genes
LINC01164
XR_946487.4
PPP2R2D
PPP2R2D
BNIP3
XR_007062339.1
ATAC
White
Beige
[0 - 737]
H3K27ac
White
Beige
[0 - 82]
H3K9me3
White
Beige
[0 - 11]
Footprints beige
CEBPG_MA1636.1
CEBPD_MA0836.2
Primers
Ctrl -

Supplementary Fig. 8. Related to Fig.5. Genome browser views of H3K27ac Hi-ChIP beige loops, ATAC, H3K27ac and H3K9me3 ChIP, C/EBP transcription factor binding sites identified by ATAC footprinting and primer location at *PDK4* (**A**) and *FABP4* (**B**) and negative control loci (**C**).

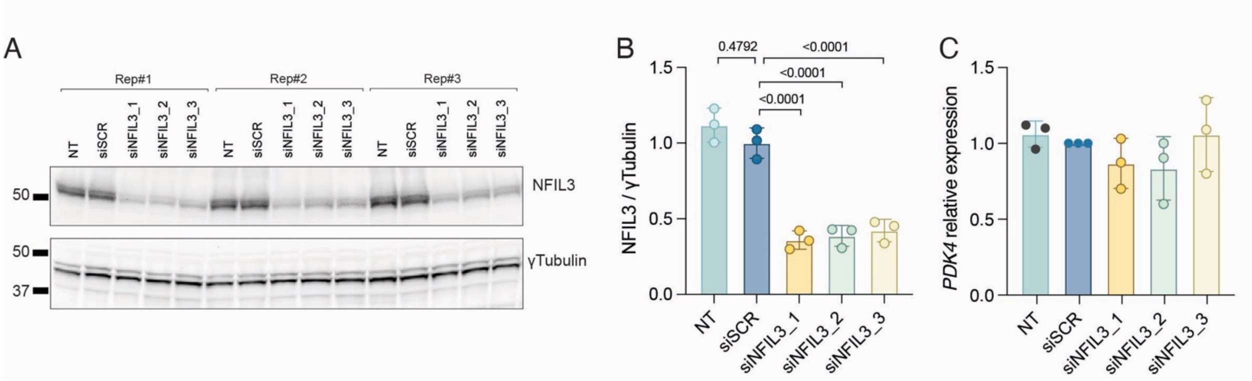

Supplementary Fig. 9. Related to Fig. 5. Western blot analysis (**A**) and quantification (**B**) of NFIL3 protein expression levels in white adipocytes 72h after transfection with one Scramble (siSCR) and 3 NFIL3-specific siRNAs (one way ANOVA; n = 3 independent differentiations). **C** RT-qPCR analysis of *PDK4* gene expression in siSCR and siNFIL3 transfected white adipocytes. NT: non-transfected.

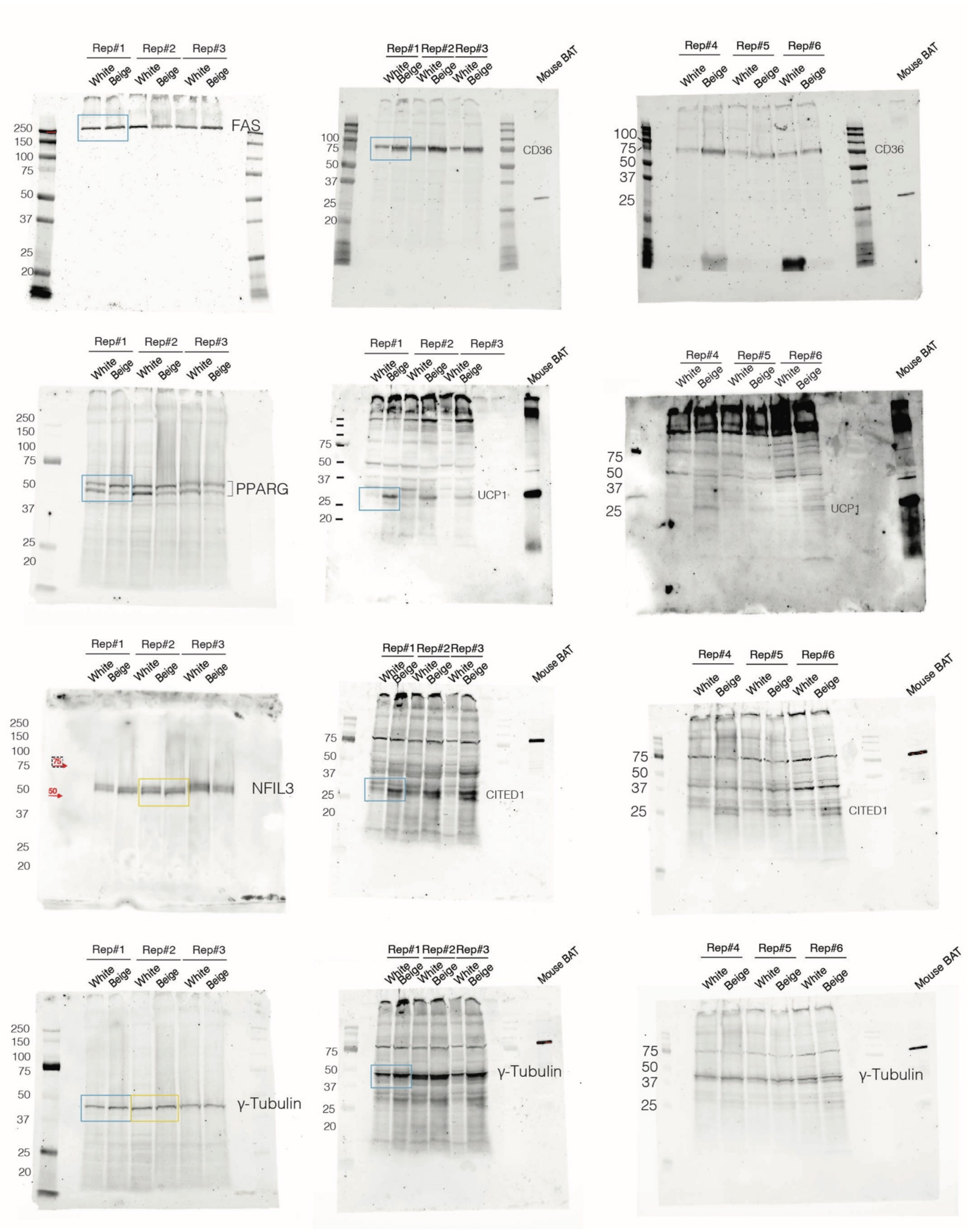

Supplementary Fig. 10. Related to Fig.1 and Supplementary Fig.7. Uncropped western blot replicates for Fig.1C and Supplementary Fig.7C.

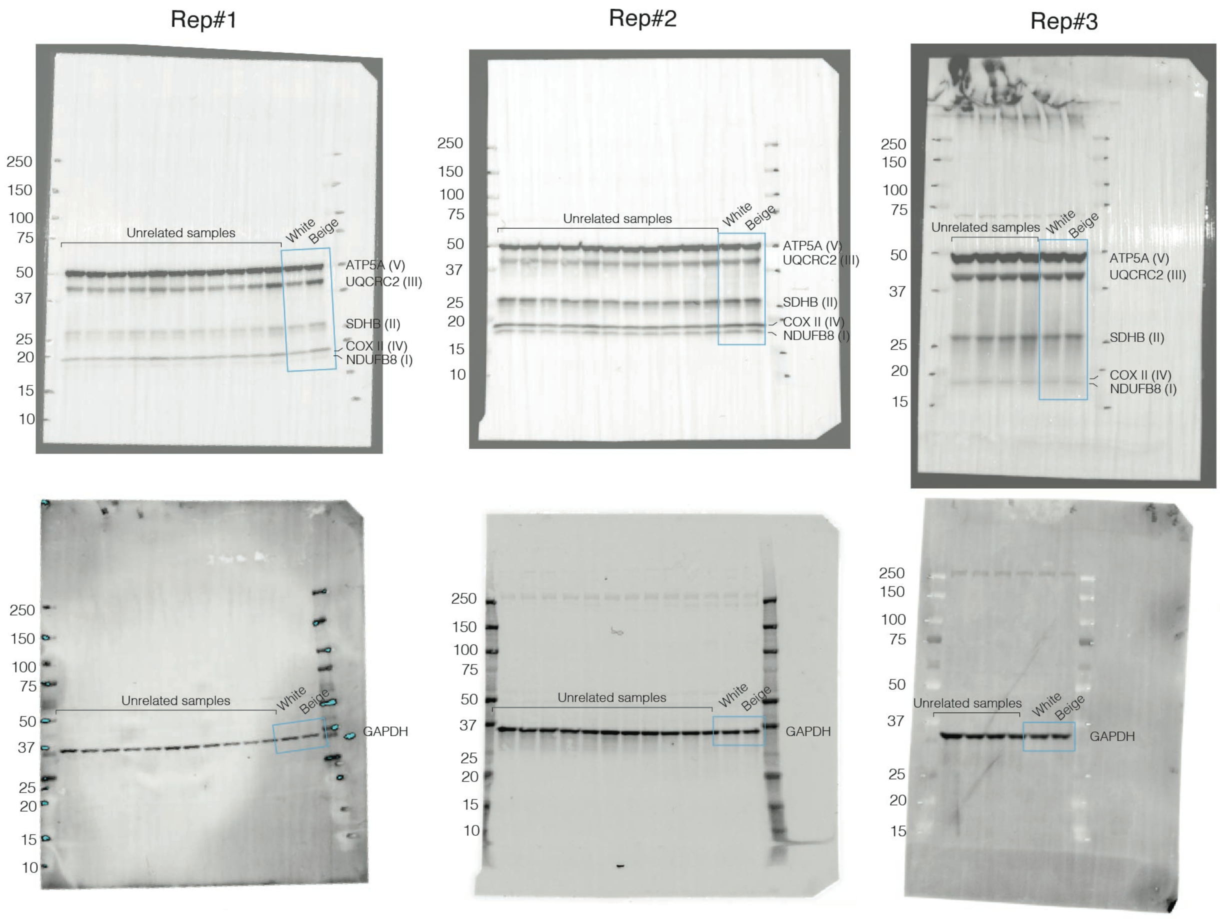

Supplementary Fig. 11. Related to Fig.1. Uncropped western blot replicates for Fig.1H.

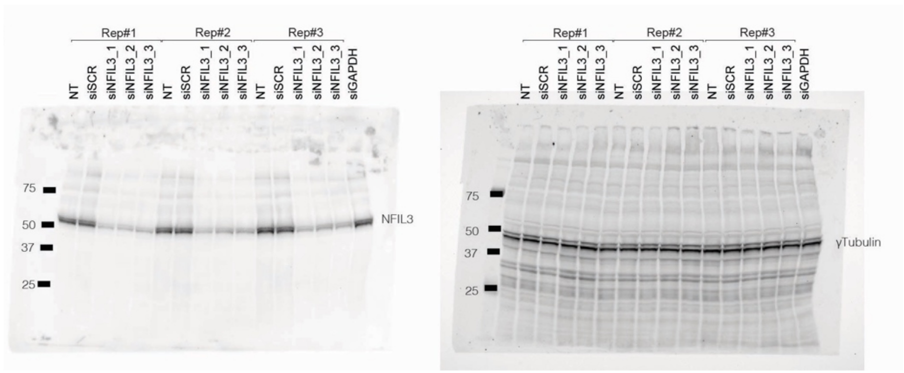

Supplementary Fig. 12. Related to Supplementary Fig.9 . Uncropped western blot replicates for Supplementary Fig.9A.
