# Supplementary material for: Multimodal epigenetic and enhancer network remodeling shape the transcriptional landscape of human beige adipocytes
Source: Commun Biol. 2026 Jan 8;9:191. doi: 10.1038/s42003-025-09469-8 (PMC12881478; doi:10.1038/s42003-025-09469-8)
Supplement: Supplementary file 3 — Description of Supplementary Data Files [file 42003_2025_9469_MOESM3_ESM.pdf]

**Multimodal epigenetic and enhancer network remodeling shape the transcriptional landscape of human beige adipocytes**

Sarah Hazell Pickering, Natalia M. Galigniana, Mohamed Abdelhalim, Anita L. Sørensen, Julia Madsen Østerbye, Manuela Zucknick, Philippe Collas, Nolwenn Briand

## Description of Additional Supplementary Data Files

**File name**: **Supplementary data 1**

**Description**: Differential gene expression between white and beige adipocytes at day15.

**File name**: **Supplementary data 2**

**Description**: ATAC peaks summary statistics (merged replicates).

**File name**: **Supplementary data 3**

**Description**: Hi-ChIP differential loop analysis (HiC-DC+).

**File name**: **Supplementary data 4**

**Description**: Differential ATAC footprinting analysis (TOBIAS).

**File name**: **Supplementary data 5**

**Description**: ChIP primers (NFIL3 ChIP-PCR).

**File name**: **Supplementary data 6**

**Description**: Source numerical data underlying the graphs presented in the figures.
