# Supplementary material for: Multimodal epigenetic and enhancer network remodeling shape the transcriptional landscape of human beige adipocytes
Source: Commun Biol. 2026 Jan 8;9:191. doi: 10.1038/s42003-025-09469-8 (PMC12881478; doi:10.1038/s42003-025-09469-8)
Supplement: Supplementary file 6 — Reporting Summary [file 42003_2025_9469_MOESM6_ESM.pdf]

nature portfolio

Corresponding author(s): Nolwenn Briand
Last updated by author(s): Sep 17, 2025

# Reporting Summary

Nature Portfolio wishes to improve the reproducibility of the work that we publish. This form provides structure for consistency and transparency in reporting. For further information on Nature Portfolio policies, see our Editorial Policies and the Editorial Policy Checklist.

## Statistics

For all statistical analyses, confirm that the following items are present in the figure legend, table legend, main text, or Methods section.

| n/a | Confirmed | |
|---|---|---|
| ☐ | ☒ | The exact sample size (*n*) for each experimental group/condition, given as a discrete number and unit of measurement |
| ☐ | ☒ | A statement on whether measurements were taken from distinct samples or whether the same sample was measured repeatedly |
| ☐ | ☒ | The statistical test(s) used AND whether they are one- or two-sided<br>*Only common tests should be described solely by name; describe more complex techniques in the Methods section.* |
| ☐ | ☒ | A description of all covariates tested |
| ☐ | ☒ | A description of any assumptions or corrections, such as tests of normality and adjustment for multiple comparisons |
| ☐ | ☒ | A full description of the statistical parameters including central tendency (e.g. means) or other basic estimates (e.g. regression coefficient) AND variation (e.g. standard deviation) or associated estimates of uncertainty (e.g. confidence intervals) |
| ☐ | ☒ | For null hypothesis testing, the test statistic (e.g. *F*, *t*, *r*) with confidence intervals, effect sizes, degrees of freedom and *P* value noted<br>*Give P values as exact values whenever suitable.* |
| ☒ | ☐ | For Bayesian analysis, information on the choice of priors and Markov chain Monte Carlo settings |
| ☒ | ☐ | For hierarchical and complex designs, identification of the appropriate level for tests and full reporting of outcomes |
| ☐ | ☒ | Estimates of effect sizes (e.g. Cohen's *d*, Pearson's *r*), indicating how they were calculated |

*Our web collection on statistics for biologists contains articles on many of the points above.*

## Software and code

Policy information about availability of computer code

| | |
|---|---|
| Data collection | For lipid droplet measurements, images were segmented with ImageJ's Morphological Segmentation plugin MorphoLibJ (https://imagej.net/plugins/morpholibj).<br>BioRad Image Lab (v6.0.0) software was used to quantify immunoblots. |
| Data analysis | - fastp v 0.20.1 was used to filter RNA-seq low-quality reads.<br>- hisat2 v2.1.0 39 was used to align RNA-seq reads<br>- featureCounts in Subread v2.0.1 was used to count RNA-seq reads<br>- Bowtie2 v2.4.5 was used to align ChIP-seq and ATAC-seq reads<br>- MACS2 v2.2.7.1 and Enriched Domain Detector (http://github.com/CollasLab/edd) were used to detect ChIP-seq peaks<br>- deepTools v3.5.3 was used to normalize ChIP-seq read counts and calculate ratio<br>- MAnorm2 v1.2.2 was used to identify differential ChIP-seq and ATAC-seq peaks<br>-fastp v0.23.2 was used to filter ATAC low quality reads<br>- Genrich v0.6.1 was used to call ATAC peaks<br>- TOBIAS v0.16.0 was used for transcription factor footprinting<br>- MAPS v2.0 was used to analyze H3K27ac Hi-ChIP datasets<br>- bwa mem v0.7.12 was used to map the raw paired-end Hi-ChIP<br>- MACS2 2.2.9.1 was used for H3K27ac Hi-ChIP peaks calling<br>- HiC-CDC+ was used to call differential Hi-ChIP loops |

For manuscripts utilizing custom algorithms or software that are central to the research but not yet described in published literature, software must be made available to editors and reviewers. We strongly encourage code deposition in a community repository (e.g. GitHub). See the Nature Portfolio guidelines for submitting code & software for further information.

## Data

Policy information about availability of data

All manuscripts must include a data availability statement. This statement should provide the following information, where applicable:

- Accession codes, unique identifiers, or web links for publicly available datasets
- A description of any restrictions on data availability
- For clinical datasets or third party data, please ensure that the statement adheres to our policy

RNA-seq data, histone ChIPs, ATAC-seq and Hi-ChIP data generated for this study are available at NCBI GEO with accession number GSE293136 and GSE256260. Re-analysis of MED1 datasets 18 can be found at GEO under accession GSE256261. Code for data processing and analysis are available at https://github.com/sarahhp/beige_3d_epigenome.

## Research involving human participants, their data, or biological material

Policy information about studies with human participants or human data. See also policy information about sex, gender (identity/presentation), and sexual orientation and race, ethnicity and racism.

| | |
|---|---|
| Reporting on sex and gender | Primary human adipose stem cells were isolated from the adipose tissue of a healthy female donor after informed consent was given. |
| Reporting on race, ethnicity, or other socially relevant groupings | N/A |
| Population characteristics | N/A |
| Recruitment | N/A |
| Ethics oversight | N/A |

Note that full information on the approval of the study protocol must also be provided in the manuscript.

# Field-specific reporting

Please select the one below that is the best fit for your research. If you are not sure, read the appropriate sections before making your selection.

☒ Life sciences ☐ Behavioural & social sciences ☐ Ecological, evolutionary & environmental sciences

For a reference copy of the document with all sections, see nature.com/documents/nr-reporting-summary-flat.pdf

# Life sciences study design

All studies must disclose on these points even when the disclosure is negative.

| | |
|---|---|
| Sample size | No sample size calculation was performed. At least 3 independent biological replicates were performed to ensure reproducibility and allow for statistical testing. |
| Data exclusions | No data were excluded from the analyses |
| Replication | Immunoblot were performed in biological triplicates<br>RNA- seq was performed in biological triplicates<br>ATAC-seq and Hi-ChIP were performed as biological duplicates<br>Histone ChIPs were performed in technical replicates and pooled replicates were sequenced. |
| Randomization | Treatment conditions were randomly assigned to parallelly  seeded differentiation experiments |
| Blinding | The investigators were not blinded during data collection or analysis. Both conditions were analyzed in a standardized way, using established pipelines. |

# Reporting for specific materials, systems and methods

We require information from authors about some types of materials, experimental systems and methods used in many studies. Here, indicate whether each material, system or method listed is relevant to your study. If you are not sure if a list item applies to your research, read the appropriate section before selecting a response.

## Materials & experimental systems

| n/a | Involved in the study |
| --- | --- |
| ☐ | ☒ Antibodies |
| ☐ | ☒ Eukaryotic cell lines |
| ☒ | ☐ Palaeontology and archaeology |
| ☒ | ☐ Animals and other organisms |
| ☒ | ☐ Clinical data |
| ☒ | ☐ Dual use research of concern |
| ☒ | ☐ Plants |

## Methods

| n/a | Involved in the study |
| --- | --- |
| ☐ | ☒ ChIP-seq |
| ☒ | ☐ Flow cytometry |
| ☒ | ☐ MRI-based neuroimaging |

# Antibodies

Antibodies used

H3K4me3 (Diagenode c15410003; Lot# A8034D)
H3K4me1 (Diagenode c15410037; Lot# A1657D)
H3K27ac (Diagenode c15410174; Lot# A.7071-001P)
H3K27me3 (Diagenode c15410069; Lot# A1818P)
H3K36me3 (Diagenode c15410058; Lot# A.8889-001P)
H3K9me3 (Diagenode c15410056; Lot# A2810P)
FAS (Santa Cruz, sc-48357; Lot# E3017)
PPARG (Thermofisher, MA5-14889; Lot# YJ40795991B)
CD36 (Santa Cruz, sc-9154; Lot# J0510)
CITED1 (Novus, H00004435-M03; Lot# L8101-5H6)
UCP1 (Abcam, 23841; Lot# GR3396557-1)
NFIL3 (Abcam, EPR27211-70; Lot#1078962-7)
γTubulin (Sigma, T5326; Lot# 080M4865)
Total OXPHOS human antibody cocktail (Abcam, ab11041; Lot# 2101031143)
GAPDH (Santa Cruz, sc-25778; Lot# D1510)

Validation

All Diagenode antibodies used are validated for human reactivity and for ChIP-seq application.

Fatty Acid Synthase Antibody (G-11; Santa Cruz, sc-48357) is a mouse monoclonal IgG1 κ antibody, raised against amino acids 2205-2504 of Fatty Acid Synthase of human origin, recommended for detection by Western blot.

PPARG (Thermofisher, MA5-14889) is a rabbit monoclonal antibody, raised against a synthetic peptide corresponding to residues surrounding Asp69 of human PPAR-gamma, recommended for detection by Western blot

CD36 Antibody (H-300; Santa Cruz, sc-9154) is a rabbit polyclonal IgG, raised against amino acids 1-300 mapping within the extracellular domain of CD36 of human origin, recommended for detection by Western blot.

CITED1 (Novus, H00004435-M03) is a mouse monoclonal IgG2a Kappa antibody, raised against a partial recombinant protein with a GST tag. Recommended for the detection of human CITED1 by Western blot

UCP1 (Abcam, 23841) is a Rabbit Polyclonal antibody. Recommended for the detection of mouse UCP1 by Western blot, and predicted to react with human. Mouse BAT loaded on the gels as a control.

NFIL3 (Abcam, EPR27211-70) is a rabbit Recombinant Monoclonal antibody. Suitable for ChIP-seq and western blot, and reacts with Human samples.

Tubulin (Sigma, T5326; mouse IgG1 isotype) is derived from the GTU-88 hybridoma produced by the fusion of mouse myeloma cells and splenocytes from BALB/c mice immunized with a synthetic peptide corresponding to a sequence the N-terminal amino acids of γ-tubulin, conjugated to KLH. The antibody reacts with human samples and is suitable for Western blot.

Total OXPHOS human antibody cocktail (Abcam, ab11041) is an optimized cocktail of antibodies for analyzing relative OXPHOS complex levels, including complexes I, II, III and IV, in Human mitochondria by Western Blot.

GAPDH (Santa Cruz, sc-25778) is a rabbit polyclonal IgG antibody, raised against amino acids 1-335 representing full length GAPDH of human origin. Suitable for Western blot.

# Eukaryotic cell lines

Policy information about cell lines and Sex and Gender in Research

Cell line source(s)

Primary human adipose stem cells were isolated from the subcutaneous adipose tissue of a healthy female donor

Authentication

None of the cells used were authenticated

Mycoplasma contamination

All cells tested negative for mycoplasma contamination

Commonly misidentified lines
(See ICLAC register)

N/A

## Plants

Seed stocks

N/A

Novel plant genotypes

N/A

Authentication

N/A

## ChIP-seq

### Data deposition

☒ Confirm that both raw and final processed data have been deposited in a public database such as GEO.

☒ Confirm that you have deposited or provided access to graph files (e.g. BED files) for the called peaks.

Data access links
*May remain private before publication.*

https://www.ncbi.nlm.nih.gov/geo/query/acc.cgi?acc=GSE293134
https://www.ncbi.nlm.nih.gov/geo/query/acc.cgi?acc=GSE256260

Files in database submission

As BED, BW, BROADPEAK/NARROWPEAK:

from GSE293134:
GSM8877153 beige_H3K27ac_D1
GSM8877154 beige_H3K27ac_D3
GSM8877155 beige_H3K27me3_D1
GSM8877156 beige_H3K27me3_D3
GSM8877157 beige_H3K36me3_D1
GSM8877158 beige_H3K36me3_D15
GSM8877159 beige_H3K36me3_D3
GSM8877160 beige_H3K4me1_D1
GSM8877161 beige_H3K4me1_D3
GSM8877162 beige_H3K4me3_D1
GSM8877163 beige_H3K4me3_D3
GSM8877164 beige_H3K9me3_D1
GSM8877165 beige_H3K9me3_D15
GSM8877166 beige_H3K9me3_D3
GSM8877167 beige_Input_D1
GSM8877168 beige_Input_D3
GSM8877169 H3K27ac_D0
GSM8877170 H3K27me3_D0
GSM8877171 H3K36me3_D0
GSM8877172 H3K4me1_D0
GSM8877173 H3K4me3_D0
GSM8877174 H3K9me3_D0
GSM8877175 Input_D0
GSM8877176 white_H3K27ac_D1
GSM8877177 white_H3K27ac_D3
GSM8877178 white_H3K27me3_D1
GSM8877179 white_H3K27me3_D3
GSM8877180 white_H3K36me3_D1
GSM8877181 white_H3K36me3_D15
GSM8877182 white_H3K36me3_D3
GSM8877183 white_H3K4me1_D1
GSM8877184 white_H3K4me1_D3
GSM8877185 white_H3K4me3_D1
GSM8877186 white_H3K4me3_D3
GSM8877187 white_H3K9me3_D1
GSM8877188 white_H3K9me3_D15
GSM8877189 white_H3K9me3_D3
GSM8877190 white_Input_D1
GSM8877191 white_Input_D3

From GSE256260:
GSM8092134 white_H3K4me3_ChIP

GSM8092135 beige_H3K4me3_ChIP
GSM8092136 white_H3K27me3_ChIP
GSM8092137 beige_H3K27me3_ChIP
GSM8092138 white_H3K27ac_ChIP
GSM8092139 beige_H3K27ac_ChIP
GSM8092140 white_H3K4me1_ChIP
GSM8092141 beige_H3K4me1_ChIP
GSM8092142 white_Input
GSM8092143 beige_Input

Genome browser session (e.g. UCSC)

https://tinyurl.com/5fxp7kad

## Methodology

Replicates

Technical duplicates were performed for each experiments and were pooled before sequencing

Sequencing depth

Paired (2x150bp) end reads at day 15 (D15) had > 42 million unique mapped reads. 75-100bp single end for additional timepoints had > 18 million uniquely mapped reads.

from GSE293134
sample total_reads q30_reads
beige_H3K36me3_D15 110 225 878 69 395 845
beige_H3K9me3_D15 113 545 326 56 731 904
H3K27ac_D0 39 710 368 27 604 173
H3K27me3_D0 40 217 022 27 629 453
H3K36me3_D0 59 985 510 44 977 810
H3K4me1_D0 48 740 629 36 304 628
H3K4me3_D0 52 854 228 30 657 931
H3K9me3_D0 54 943 071 36 400 490
input_D0 46 976 642 25 652 485
white_H3K27ac_D1 43 932 244 26 114 489
white_H3K27ac_D3 39 776 832 25 695 266
white_H3K27me3_D1 26 963 359 18 672 365
white_H3K27me3_D3 34 313 341 24 951 121
white_H3K36me3_D1 44 441 712 33 110 975
white_H3K36me3_D15 61 449 282 42 355 688
white_H3K36me3_D3 45 936 057 34 719 900
white_H3K4me1_D1 48 920 851 37 349 158
white_H3K4me1_D3 44 199 282 34 124 171
white_H3K4me3_D1 52 400 593 29 676 657
white_H3K4me3_D3 48 741 122 28 268 298
white_H3K9me3_D1 47 781 467 31 018 262
white_H3K9me3_D15 93 986 226 49 798 458
white_H3K9me3_D3 52 019 763 33 657 469
white_input_D1 34 561 242 21 755 465
white_input_D3 42 551 012 25 207 675
beige_H3K27ac_D1 78 533 092 72 751 767
beige_H3K27ac_D3 76 124 306 69 901 696
beige_H3K27me3_D1 58 789 504 49 613 245
beige_H3K27me3_D3 60 978 338 51 493 152
beige_H3K36me3_D1 59 064 852 52 992 116
beige_H3K36me3_D3 101 640 512 90 436 763
beige_H3K4me1_D1 61 003 784 56 944 775
beige_H3K4me1_D3 104 353 626 96 743 521
beige_H3K4me3_D1 104 127 676 95 845 068
beige_H3K4me3_D3 102 199 994 93 510 973
beige_H3K9me3_D1 42 906 052 32 527 108
beige_H3K9me3_D3 77 511 278 59 362 743
beige_Input_D1 78 144 492 67 999 734
beige_Input_D3 61 033 832 52 259 436

from GSE256260 (all D15)
sample total_reads q30_reads
white_H3K27ac_D15 90 847 264 60 623 428
white_H3K27me3_D15 121 624 184 71 546 332
white_H3K4me1_D15 143 883 078 88 662 942
white_H3K4me3_D15 189 565 822 85 971 949
white_input_D15 110 436 496 82 659 353
beige_H3K27ac_D15 258 555 086 98 962 452
beige_H3K27me3_D15 139 378 028 74 444 675
beige_H3K4me1_D15 128 346 764 83 710 487
beige_H3K4me3_D15 224 134 376 82 284 997
beige_input_D15 110 937 246 84 278 841

Antibodies

H3K4me3 (Diagenode c15410003; Lot# A8034D)
H3K4me1 (Diagenode c15410037; Lot# A1657D)
H3K27ac (Diagenode c15410174; Lot# A.7071-001P)
H3K27me3 (Diagenode c15410069; Lot# A1818P)
H3K36me3 (Diagenode c15410058; Lot# A.8889-001P)
H3K9me3 (Diagenode c15410056; Lot# A2810P)

Peak calling parameters

FASTQ sequences from H3K4me3, H3K27ac, H3K4me1, H3K36me3, H3K27me3 and H3K9me3 ChIPs were aligned to hg38 using Bowtie2 v2.4.5. Peaks were detected using MACS2 v2.2.7.1, except for H3K9me3 ChIP where peaks were called using Enriched Domain Detector (http://github.com/CollasLab/edd) with gap penalty and bin size defined as the mean outputs of 10 runs in auto-estimation mode. Differential peaks were identified using MAnorm2 v1.2.2 with default parameters.

Data quality

from GSE293134
sample peaks_passing_filters
beige_H3K27me3_D1 57378
beige_H3K9me3_D1 821
H3K27ac_D0 112032
beige_H3K36me3_D1 113241
white_H3K27ac_D3 80552
beige_H3K4me3_D3 66198
white_H3K27ac_D1 106321
H3K9me3_D0 322
beige_H3K27ac_D3 212691
white_H3K9me3_D1 295
beige_H3K4me1_D3 211620
white_H3K9me3_D3 272
beige_H3K27me3_D3 61495
beige_H3K9me3_D3 1002
beige_H3K36me3_D3 123195
H3K27me3_D0 40065
white_H3K27me3_D3 73848
white_H3K27me3_D1 54712
H3K4me1_D0 125234
white_H3K4me1_D3 139708
white_H3K4me1_D1 114696
white_H3K9me3_D15 423
beige_H3K9me3_D15 412
white_H3K36me3_D15 106253
beige_H3K36me3_D15 97733
H3K4me3_D0 31895
white_H3K4me3_D3 31071
H3K36me3_D0 73872
white_H3K4me3_D1 30110
white_H3K36me3_D1 38128
beige_H3K4me3_D1 64558
white_H3K36me3_D3 70666
beige_H3K27ac_D1 226550
beige_H3K4me1_D1 175114

from GSE256260 (all D15)
sample peaks_passing filters
white_H3K4me3_ChIP 24166
beige_H3K4me3_ChIP 26582
white_H3K27me3_ChIP 106112
beige_H3K27me3_ChIP 103752
white_H3K27ac_ChIP 104716
beige_H3K27ac_ChIP 65094
white_H3K4me1_ChIP 177983
beige_H3K4me1_ChIP 178484

Software

- Bowtie2 v2.4.5 was used to align ChIP-seq reads
- MACS2 v2.2.7.1 and Enriched Domain Detector (http://github.com/CollasLab/edd) were used to detect ChIP-seq peaks
- deepTools v3.5.3 was used to normalize ChIP-seq read counts and calculate ratio
- MAnorm2 v1.2.2 was used to identify differential ChIP-seq peaks
